# Preparing for Flight

**Published:** 1891-07-25

**Authors:** 


### THE DOCTOR'S ARMCHAIR.

### PREPARING FOR FLIGHT.

THE birds, in some respects, are the luckiest of all creatures. They take their periodical holidays apparently without a moment's consideration whenever the mood comes upon them. "Where to go," is a question which often drives a middle-class man to despair. There are so many interests to be consulted; wife, and children, pocket, pleasure, health. These are considerations which can neither be set aside nor treated lightly. Fancy the effect of trying to treat your wife lightly! Every husband may draw his own picture of the consequences. The pocket, too, is a most serious consideration in these times of scarcity. Many of us live at the very top of our income; and even when we have done that we find ourselves at such a very inconsiderable elevation that we make all sorts of efforts to climb to a much higher point. But this leaves little for the summer holiday, though the holiday, to be a holiday at all, cannot be taken on a little. Health, moreover, is a tremendously practical question nowadays. To the husband it is the very essence of continued existence. If he is not well he cannot do his best work; and if he cannot do his best work he falls behind Smith and Brown and other fellows who are running the race of business with him, and who, in many respects, are much inferior to himself. He must have health at whatever cost, or himself, his wife, his family, and, indeed, his whole earthly establishment will collapse. That is a thought not to be endured.

The birds have none of these preliminary problems to solve. When they want to go off for change of air, they go; and they go exactly where they please. They do not require to seek out a policeman "without encumbrance" to take care of the house in their absence; they pack no trunks and band boxes; nor do they send for a two horse 'bus to carry them and their household gods to the station. They have no bargains to make with greedy landladies and no enquiries to institute about the sanitary condition of their temporary summer home. They make up their minds; they fly across the seas; they alight in their new territory; they build themselves houses and rear their families; and they enjoy themselves without thought or care the livelong day. Enviable creatures!

The middle of July has once more come and gone, and we have actually had a few days of summer at last. Of spring, in London at any rate, we had none. Does it strike readers how very sunless a period we Londoners have experienced ever since the beginning of last November? The present writer can remember nothing like it for the past fifteen years. But the sun is one of the primest of all the essentials of life, health, and strength. Without his presence for a sufficient number of hours each day, health is impossible. A sunless London means a deadly London; and the influenza, with a variety of chest diseases, have had much the same effect upon us during the last few months as a minor pestilence. Those of us who have had the influenza this season are uncommonly "seedy." We have hardly any nerve left; there is no "go" in us; we are "weary, flat, stale, and unprofitable" in an extraordinary and most disgusting degree. For us a lengthened migration to more favourable climes is absolutely indispensable.

But the languor and feebleness that come with the summer are not confined to the influenza convalescent. The niggardly sun has been so sparing of his smiles and his gifts of health that all of us together are limp and washed out. As a fish thrown out upon the bank gasps for the air which he can only get from water, so we gasp for the stimulating ozone which for us can only be found in quantity on the mountain or by the sea. This limp condition is as positively bad as it is unendurable to the sensibilities. It is to be got rid of at all costs. No man can afford to face the winter with it. The summer migration is as essential to the Londoner as the spring and autumn flights are to the migratory birds.

Somebody has said that no man who is over forty and under sixty years of age should walk less than seven or eight miles a day: that is, of course, on the assumption that walking is his only exercise. It is doubtful if many of us walk anything like seven or eight miles a day when we are in the full tide of business; but we should take care to walk that and a good deal more when we are out of town on our annual holiday. It, therefore, becomes a matter of importance to choose a place where there are plenty of breezy, bracing, and pleasant walks. In this particular, a mountainous region usually has advantages over a seaside place. At the seaside there is only half the circumference of a circle within which to perambulate; the other half is occupied by the rising and falling waters. In many other respects, too, the mountain is to be preferred to the sea; the air, as a rule, is quite as pure, and it is much more stimulating and light. Whilst there is, generally speaking, no doubt about the purity of the air at the seaside, it cannot be denied that it is often wanting in dryness and lightness. For the man who lives in the fog and smoke and the dense atmosphere of London ten or eleven months in the year, nothing can be so great a change and so entirely valuable as the light bracing and stimulating air of the mountains.

The majority of people, it must be admitted, prefer the seaside to the country or the hills. There are seldom any "nigger" minstrels in the country, or any of the other thousand-and-one cockney attractions that seaside places can boast. If the sea be chosen it is best to select a place that has a good background of hills, so that long walks may be taken, and high atmospheric levels reached and lived in for at least a few hours every day. Next to food, pure bracing air and exercise are the most essential of all the conditions of health. This is an exceedingly stale old truism, but it will have to be repeated again and again, like the parson's sermon on repentance, until people actually lay it to heart, and carry it out in practice. The business of gaining and maintaining good health is much more arduous than many people imagine. Few persons give a thousandth part of the thought to health which they give to the acquisition of wealth. But in these days of keen brain struggle and patient waiting for fortune health is wealth. Before the world is fifty years older, unless some grand cataclysm takes place, the healthiest man will invariably be the wealthiest man. He, and she too, who is now taking thought and making preparation for the summer holiday had better lay some of these homely considerations to heart.

